# Evaluation of Topical Antifungals Using a New Predictive Animal Model for Efficacy against Severe Tinea Unguium: A Comparison of Efinaconazole and Luliconazole

**DOI:** 10.1007/s11046-022-00664-3

**Published:** 2022-09-12

**Authors:** Akane Masumoto, Keita Sugiura, Yoshiki Matsuda, Haruki Tachibana, Yoshiyuki Tatsumi

**Affiliations:** 1 Pharmacology Department, Drug Research Center, Kaken Pharmaceutical Co., Ltd., 14 Shinomiya, Minamigawara-cho, Yamashina-ku, Kyoto, 607-8042 Japan; 2 Pharmacokinetics and Safety Department, Drug Research Center, Kaken Pharmaceutical Co., Ltd., 301 Gensuke, Fujieda-shi, Shizuoka, 426-8646 Japan

**Keywords:** Arthrospore, In vivo model, Mycology, Severe onychomycosis, Topical antifungal drug

## Abstract

Development of new topical drugs requires an animal onychomycosis model that can predict the drug efficacy against moderate to severe human onychomycosis because the severity of onychomycosis varies and affects the drug efficacy. This study established a non-immunosuppressive guinea pig tinea unguium model under 8-week infection condition in addition to a previously reported model under 4-week infection condition. In the tinea unguium model, most fungi were tightly present in the arthrospore form, like in human onychomycosis. The topical formulations of efinaconazole and luliconazole, two azole class anti-onychomycosis drugs, were evaluated for their efficacy in these models. In the untreated group, the nail fungal burden in the 8-week model was higher than that in the 4-week model and the stronger infection intensity affected the efficacy of the drugs, suggesting that the 8-week model was more severe. The 90% efficacy rate (42%) of luliconazole in the 8-week model was significantly lowered than that (83%) in the 4-week model, and its 99% efficacy rates were 0% in both models. Conversely, the 90% and 99% efficacy rates of efinaconazole (92% and 50% in the 4-week model, and 75% and 25% in the 8-week model, respectively) were not significantly different between the two infection durations. In addition, efinaconazole was more effective than luliconazole in reducing the nail fungal burden. Considering the relevance of clinical reports of the effectiveness of efinaconazole on severe onychomycosis, the new severe tinea unguium model would predict drug efficacy against moderate to severe onychomycosis.

## Introduction

Onychomycosis is an infectious disease primarily caused by *Trichophyton rubrum* and *Trichophyton interdigitale* and has an estimated prevalence of 10% in Japan and 13.8% in the USA [1, 2]. Generally, onychomycosis is difficult to cure completely and reduces the patient’s quality of life due to difficulty in walking and poor nail appearance [3] as well as causes secondary infections to other family members [4]. Distal lateral subungual onychomycosis (DLSO), among several types of onychomycosis, is reported to be the most common type [5, 6]. The infection involves the subungual space and nail matrix in DLSO, extending proximally in advanced cases. A couple of drugs, oral and topical antifungals, are used for treating onychomycosis [7].

Nevertheless, the severity of onychomycosis in humans varies and affects the drug efficacy. Furthermore, the complete cure rates of topical antifungals against severe onychomycosis are generally lower than those of oral drugs [7], and the development of new topical drugs with advanced efficacy is desired. Thus, an animal onychomycosis model with increased infection intensity is essential to predict the drug potency in severe human onychomycosis. However, there have been no reports on the influence of infection intensity on drug potency in the animal onychomycosis model.

Generally, the in vivo efficacy of anti-onychomycosis drugs is evaluated using guinea pig or rabbit models [8–12]. In the production of several onychomycosis models, animals were treated with immunosuppressants to establish infection [9, 10]. Other models have shown stable nail infection in animals without immunosuppression [8, 11, 12]. The morbidity risk of onychomycosis has been reported to be higher in patients receiving long-term administration of immunosuppressive drugs than in healthy people [13], indicating the involvement of the host defense ability in the fungal pathogenesis. Notwithstanding, onychomycosis is a common infection among healthy people [13], and hence a non-immunosuppressed animal model would be more appropriate for drug efficacy evaluation. It has been reported that the host immunity is concerned with the disease state formation, affecting the growth form of the fungus and localization in the nails [13, 14]. In addition, the most common pathogen of human onychomycosis is *T. rubrum* [1], but there have been no reports on establishing animal tinea unguium models using *T. rubrum*. *Trichophyton rubrum* is adapted to humans [5] and does not naturally infect animals [15]. Thus, zoophilic *T. mentagrophytes* is commonly used to produce experimental animal tinea unguium.

This study established a non-immunosuppressed tinea unguium model with a stronger infection intensity than the previously reported model (4-week infection) [11, 12] by extending the infection duration to 8 weeks. Furthermore, efinaconazole and luliconazole, two azole class topical anti-onychomycosis drugs with different nail permeabilities and keratin affinities [10, 16], were evaluated for their efficacy in the tinea unguium models to investigate the effect of the infection duration on therapeutic efficacy of the topical drugs. In addition, to consider the extractability of the animal model to human onychomycosis, in vitro antifungal activities of the drugs against the arthrospores (parasitic form) of *T. mentagrophytes* and *T. rubrum*, were evaluated.

## Materials and Methods

### Test Substances

Efinaconazole (Sigma-Aldrich Co., LLC., St. Louis, MO, USA) and luliconazole (Toronto Research Chemicals, Toronto, ON, Canada) were used for the minimum effective concentration (MEC) test. Furthermore, to evaluate the therapeutic efficacy in guinea pig tinea unguium models, we used efinaconazole 10% solution (Kaken Pharmaceutical, Co., Ltd., Tokyo, Japan) prescribed clinically in Asia and North America, including Japan [10], and luliconazole 5% solution (Sato Pharmaceutical Co., Ltd., Tokyo, Japan) prescribed clinically in Japan for human onychomycosis treatment [10].

### Strains

*T. mentagrophytes* SM-110 strain was provided by the Niigata University School of Medicine (Niigata, Japan). *T. rubrum* IFM 46157 strain was obtained from the Medical Mycology Research Center, Chiba University (Chiba, Japan).

### Preparation of Arthrospores of *T. mentagrophytes *and *T. rubrum*

The arthrospores were prepared using a carbon dioxide culture medium according to the method of Fujita et al. [17]. Shortly, a membrane filter (pore size: 0.45 μm) was placed on a brain–heart infusion agar medium (Nissui Pharmaceutical Co., Ltd., Tokyo, Japan), onto which 100 μL of each strain’s microconidial suspension was added and plated. The agar plate was placed in an airtight container, generating carbon dioxide (theoretical concentration: 17%), and cultured at 30 °C for *T. mentagrophytes* and 35 °C for *T. rubrum* for 10 days. The arthrospores grown on the filter were collected and suspended in saline containing 0.05% Tween 80, giving fungal concentrations of 10^6^ and 10^8^ cells/mL for the MEC test and the production of the guinea pig tinea unguium models, respectively. For the MEC test, *T. mentagrophytes* and *T. rubrum* were used, and for the production of the animal models, *T. mentagrophytes* was used.

### In vitro Antifungal Activity Against the Arthrospores

Antifungal activity was determined as follows: 200 mg of sterilized keratin powder (prepared by the method previously reported [11]) was added to the test tube as a condition for adding 20% keratin, and 500 μL of 3-(N-morpholino) propanesulfonic acid buffered RPMI 1640 medium (Nissui Pharmaceutical Co., Ltd., Tokyo, Japan) containing the test material (prepared by a two-fold stepwise dilution) and 500 μL of fungal suspension (final concentration: 10^4^ cells/mL) were added after sterilizing the keratin powder. The mixture was statically incubated at 35 °C for 7 days. Generally, the antifungal activity is evaluated using the minimum inhibitory concentration (MIC), which is determined visually. However, in this study, the arthrospores were precipitated in a liquid medium, making it difficult to determine the MIC visually. Thus, the MEC was determined by microscopic examination of the fungi in the culture media and defined as the lowest drug concentration at which no obvious germination or hyphal elongation was observed compared with the growth control. The geometric mean MEC was calculated for each strain of the two independent experiments performed.

### The Guinea Pig Tinea Unguium Model

#### The Production of the Guinea Pig Tinea Unguium Model and Histopathological Examination of Infected Nails

**Fig. 1:**
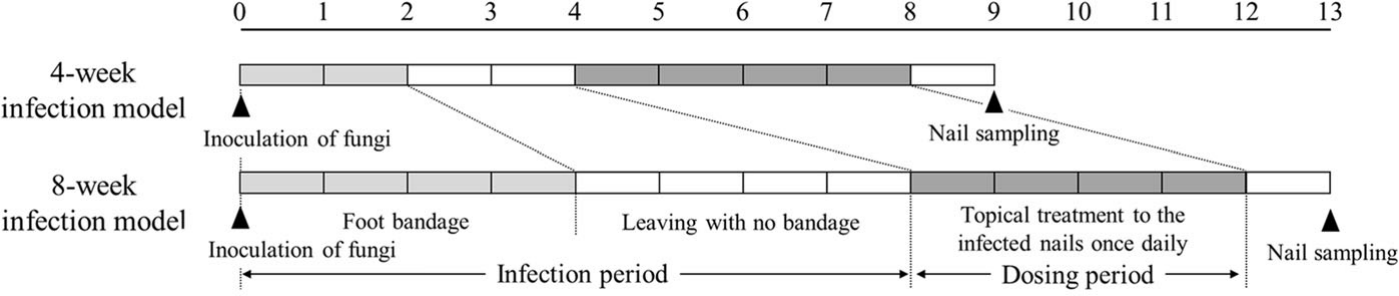
The production schedule of the guinea pig tinea unguium model

The arthrospore suspensions of *T. mentagrophytes* (inoculum size: 2 × 10^7^ cells/foot) were inoculated into the plantar skin and between the toes (between the second and third toes and between the third and fourth toes) of the guinea pigs’ hind paws, as previously reported [11, 12]. The experiments were performed with a group of six animals (12 feet). The dermatophyte was transmitted from the skin to the nails by a 28-day (4-week) or 56-day (8-week) infection period, resulting in the production of the tinea unguium model (Fig. 1). A portion of the hind paw nails was collected in the 4-week infection model before the treatment. The morphology of the fungi in the nails was observed by light microscopy after the nail dissolution with 20% KOH. Before the 4-week infection treatment, the nail pieces of guinea pigs were collected, fixed in 10% neutral buffered formalin solution, and then demineralized with 10% formic acid–citric acid solution for 4 weeks (the fluid exchanges every 1 week). After demineralization, the nails were excised and neutralized with an aqueous sodium sulfate solution overnight. Eventually, paraffin embedding, slicing, and Grocott’s staining were performed using the conventional method. The nail specimens were then observed under a light microscope.

#### Evaluation of Therapeutic Efficacy in the Guinea Pig Tinea Unguium Models

Efinaconazole 10% solution or luliconazole 5% solution was applied to the nails of the hind paw once daily for 28 consecutive days. Each treatment was initiated on the 29th or 57th day after the arthrospore suspension inoculation. All animals were euthanized one week after the previous treatment. For one group, six animals with 12 feet were used. Their nails were collected per foot and homogenized with a glass homogenizer in PBS (pH 7.4) containing 0.25% trypsin and digested at 37 °C for about 1 h. A portion of the homogenate was dropped onto modified glucose peptone agar with lecithin and polysorbate 80 containing 1% lecithin and antibiotics. Then, it was plated and cultured at 30 °C for 14 days. The number of fungal colonies on the agar was used to calculate the number of viable fungi in the nails. The percentage of nails showing 90% or more and 99% or more viability decrement compared to the average number of fungi in the foot nails of the infected control group was calculated in each group (total 12 nail samples from 12 feet) and was defined as 90% and 99% efficacy rates, respectively. The Student’s t-test (one-tailed) was carried out at a significance level of 95% to compare viable fungal counts in the nails of infected controls in 4- and 8-week infection models. The Fisher’s exact test (one-tailed) was performed at a significance level of 95% for the differences in the efficacy rates of drugs in infection periods. JMP^®^ 16 (SAS Institute Inc., Cary, NC, USA) was used for the analyses.

#### Measurement of Drug Concentration in Nails

After the drug was extracted by adding methanol to the nail homogenate, the drug concentration in the homogenate extracts (total 12 nail samples from 12 feet) were measured using liquid chromatography-tandem mass spectrometry (LC–MS/MS). The drug concentrations in the nail were calculated (μg/g nail). Then, the Student’s t-test (two-tailed) was performed at a significance level of 95% for the differences in the drug concentrations between treatment groups. JMP^®^ 16 (SAS Institute Inc., Cary, NC, USA) was used for the analysis.

## Results

### In vitro Antifungal Activity Against Arthrospores of *T. mentagrophytes *and *T. rubrum*

The geometric mean MECs of efinaconazole for arthrospores of *T. mentagrophytes* and *T. rubrum* in RPMI 1640 medium with 20% keratin were 0.13 and 0.25 μg/mL, respectively (Table 1). The geometric mean MECs of luliconazole for these arthrospores were 0.13 and 0.25 μg/mL, respectively. Thus, the antifungal activities of the two drugs against arthrospores of the species were almost identical under 20% keratin condition.

**Table 1:** In vitro antifungal activities of the drugs against arthrospores in the presence of 20% keratin

Drugs	MEC^a^ in a medium with keratin (µg/mL)	
	*T. mentagrophytes* SM-110	*T. rubrum* IFM 46157
Efinaconazole	0.13	0.25
Luliconazole	0.13	0.25

^a^MEC (minimum effective concentration) was represented as a geometric mean value of two independent experiments

### KOH Direct Microscopy and Histopathological Observation of the Infected Nails

**Fig. 2:**
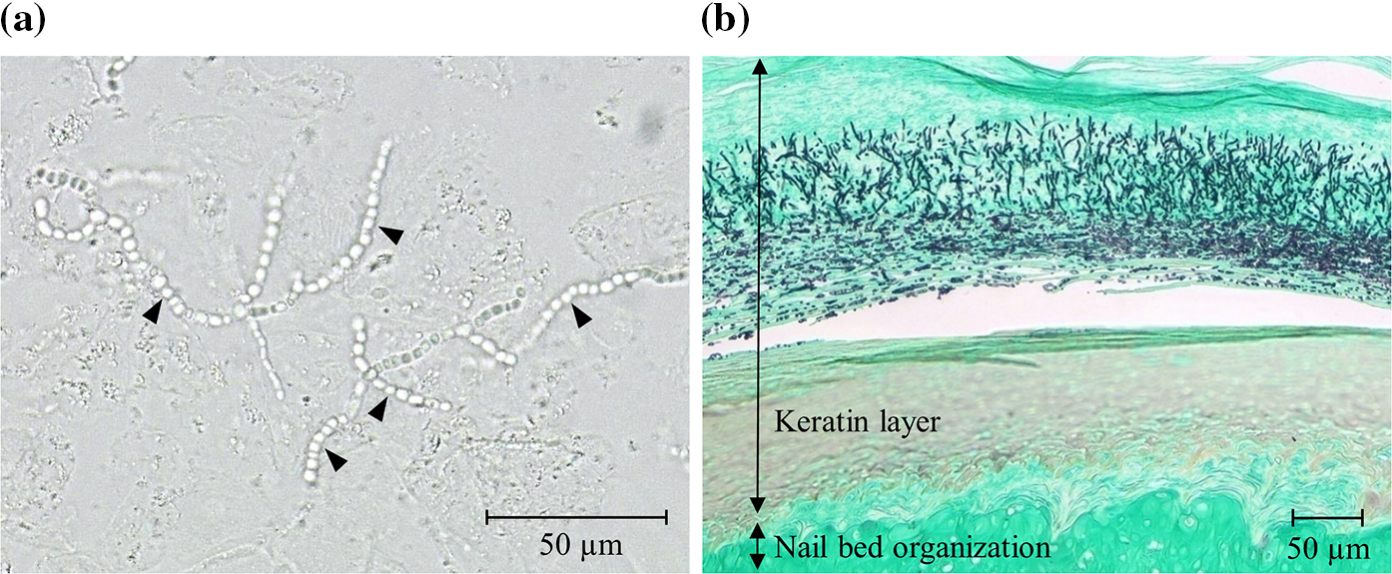
*T. mentagrophytes* infected guinea pig nails. **a** KOH direct microscopic image: before the treatment, in the 4-week infection model, a part of the nails was lysed with 20% KOH, and the morphology of fungi in the nails was observed by light microscopy. Arrowheads indicate the observed fungi. **b** Histopathological observation: nail pieces were collected before the treatment in the 4-week infection model. Thin sections and Grocott’s staining were performed after fixation and demineralization and observed under a light microscope

Before treatment, many *T. mentagrophytes* were tightly present in the form of arthrospores in the nails in the 4-week infection model (Fig. 2a). Furthermore, at most sites of infection, many fungi (arthrospores and hyphae) infected the inside of the nail’s keratin layer (Fig. 2b).

### Nail Fungal Burden of Infected Control Groups in 4-week and 8-week Infection Models

The viable fungal counts in the nails of the infected control group in 4- and 8-week infection models after the treatment period were 4.60 ± 0.26 and 4.94 ± 0.30 Log colony-forming unit in the nails/foot (mean ± SD), respectively. The number of viable fungi in the nails in the 8-week infection model was 2.3 times higher than that in the 4-week infection model, with a statistically significant difference (*p* = 0.0062).

### Therapeutic Efficacy of Drugs in Both Infection Models

**Fig. 3:**
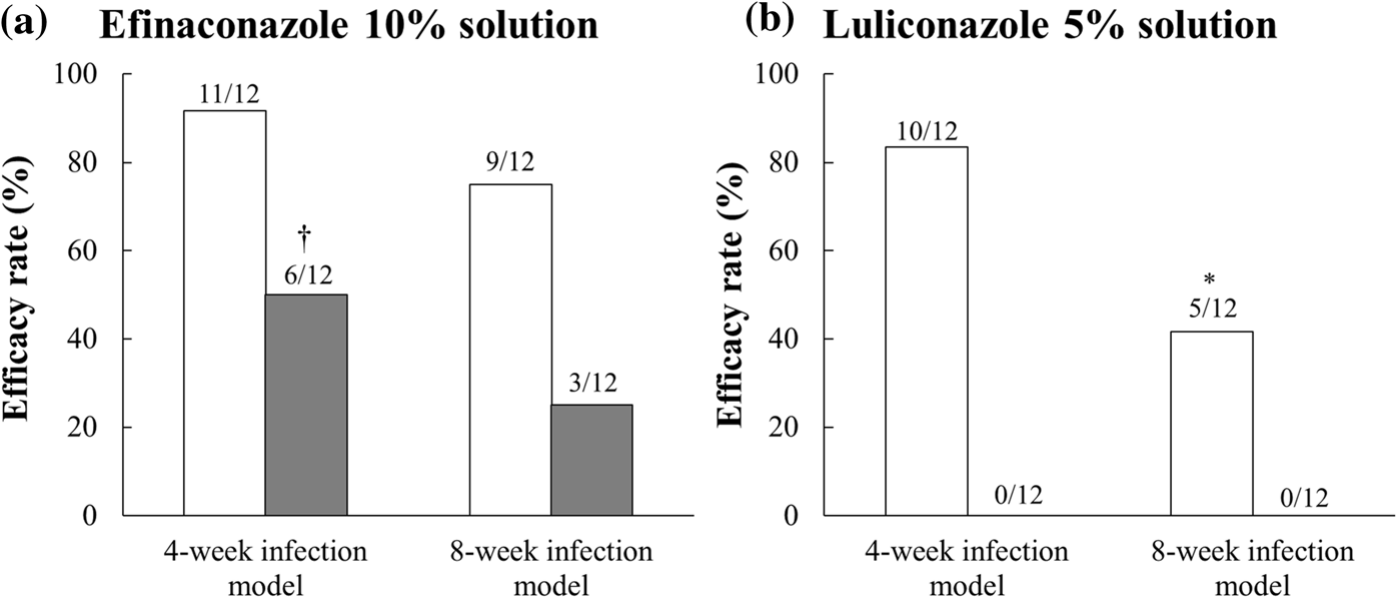
Efficacy rates of efinaconazole 10% solution and luliconazole 5% solution in guinea pig tinea unguium models. Nails were collected 1 week after the last treatment and homogenized using a glass homogenizer. The viable fungal count in the nails was calculated from the counts of colony-forming units present in the nail homogenates. The rates of nails showing 90% and 99% efficacy in each group (90% and 99% efficacy rates, respectively) were calculated. Ninety percent and 99% efficacy were defined as 90% or more and 99% or more reduction, respectively, in the viable fungal count compared to the average viable fungal count in the infected control group (12 nails per group). White bars indicate 90% efficacy rate, and gray bars indicate 99% efficacy rate. The numbers above the bars indicate the number of validated cases/total number of cases. *: *p* < 0.05 vs. the 4-week infection model (Fisher’s exact test). †: *p* < 0.05 vs. the luliconazole 5% solution (Fisher’s exact test)

The 90% efficacy rates of the efinaconazole 10% solution groups in the 4- and 8-week infection models were 92% (11/12) and 75% (9/12), respectively (Fig. 3a). There was no statistically significant difference between the two infection models (*p* = 0.2950). The 99% efficacy rates of the efinaconazole 10% solution groups in the 4- and 8-week infection models were 50% (6/12) and 25% (3/12), respectively. There was no statistically significant difference between the two infection models (*p* = 0.2002). In contrast, the 90% efficacy rates of the luliconazole 5% solution groups in the 4- and 8-week infection models were 83% (10/12) and 42% (5/12), respectively (Fig. 3b), with a statistically significant difference between the two infection models (*p* = 0.0447). The 99% efficacy rates of the luliconazole 5% solution groups in 4- and 8-week infection models were both 0% (0/12), and the statistical analysis was not performed.

In the 4-week infection model, there was no statistically significant difference between the 90% efficacy rates of efinaconazole 10% solution and luliconazole 5% solution groups (*p* = 0.5000), whereas the 99% efficacy rate of efinaconazole 10% solution was significantly higher than that of luliconazole 5% solution (*p* = 0.0069). In the 8-week infection model, the 90% and 99% efficacy rates of efinaconazole 10% solution were higher than those of luliconazole 5% solution (*p* = 0.1069 and *p* = 0.1087, respectively).

### Drug Concentration in the Nails

In the 4-week infection model, the drug concentrations (mean ± SD) in the nails of efinaconazole 10% solution and luliconazole 5% solution groups were 1720 ± 1400 and 3290 ± 1040 μg/g nail, respectively (Table 2), with a statistically significant difference between the two drugs (*p* = 0.0049). In the 8-week infection model, the drug concentrations in the nails of efinaconazole 10% solution and luliconazole 5% solution groups were 3080 ± 1360 and 4160 ± 3160 μg/g nail, respectively (Table 2), with no statistically significant difference between the two drugs (*p* = 0.2878). There was a statistically significant difference between the concentrations of efinaconazole in the infection periods (*p* = 0.0242). In contrast, there was no statistically significant difference between luliconazole groups (*p* = 0.3738).

**Table 2:** Drug concentrations in the nails 1 week after the last treatment

Groups	Total drug concentration (mean ± SD, µg/g nail)	
	4-week infection model	8-week infection model
Efinaconazole 10% solution	1720 ± 1400	3080 ± 1360^*^
Luliconazole 5% solution	3290 ± 1040^††^	4160 ± 3160

^*^p < 0.05 vs. the 4-week infection model (Student’s t-test)

^††^*p* < 0.001 vs. efinaconazole 10% solution (Student’s t-test)

## Discussion

Dermatophytes form arthrospores in the human nails and the stratum corneum of the human skin [18]. Arthrospore formation is believed to be primarily responsible for dermatophyte infection and transmission in humans [19]. In this study, animal tinea unguium models without immunosuppression were produced because the host immunity is concerned with the disease state formation, affecting the growth form of the fungus and localization in the nails [13, 14]. The fungal form and localization in the model have not been clarified in detail, and those were histopathologically examined in this study. The histopathological observation showed that most fungi in the nails of the guinea pig model were present in an arthrospore form, as reported in human nails [18]. Moreover, several fungi infected the nail plate rather than the nail’s surface. The pathological results of the nail were close to the finding, “sandwich sign” [20]; the fungus was confirmed between two zones of the cell layers in the stratum corneum of the patients with skin dermatophytosis. Therefore, this tinea unguium model was considered to be similar to a clinical-pathological condition in the infection form and dermatophyte localization. In addition, the arthrospores of *T. mentagrophyte*s and *T. rubrum* were susceptible to efinaconazole and luliconazole at almost the same level, as reported in their susceptibilities to several other antifungals [21]. From these findings, the efficacy of drugs in this animal model using *T. mentagrophytes* was considered relevant to the clinical efficacy against human onychomycosis, primarily caused by *T. rubrum*.

Furthermore, since the efficacy of drugs on onychomycosis is affected by its severity, an animal onychomycosis model that can predict their clinical efficiency against moderate to severe human onychomycosis is deemed valuable. Therefore, a predictive guinea pig tinea unguium model was newly produced under the conditions discussed above with a stronger infection intensity than a previously reported model (4-week infection) [11, 12] by extending the infection duration to 8 weeks. As a result, in the infected control group, the viable fungal count in the nails of the 8-week infection model was significantly higher than that of the 4-week infection model, which implied that the fungi would have spread in the nails after 8 weeks of infection. Efinaconazole and luliconazole, which have the same mechanisms of action but different nail permeabilities and keratin affinities [10, 16], were evaluated in the models with different infection duration to investigate the effects of infection duration on the therapeutic efficacy of the drugs. The mean drug concentration in the nail of the 8-week infection model was slightly higher for luliconazole and siginificantly higher for efinaconazole than that in the nail of the 4-week infection model. The nail infection progression in the 8-week infection model compared to the 4-week infection model was speculated to reduce the drug penetration barrier function in the nail, resulting in an increase in the drug concentration in the nail. However, their efficacy against the tinea unguium model was lower in the 8-week infection model than in the 4-week infection model. These results suggested that the 8-week infection model is more resistant to topical antifungal therapy than the 4-week infection model because of its higher fungal burden.

Efinaconazole 10% solution showed therapeutic effects on the 4- and 8-week infection models (90% efficacy rates: 92% and 75%, 99% efficacy rates: 50% and 25%, respectively). Its efficacy rates in the 8-week infection model were slightly reduced compared with those in the 4-week infection model, but there was no statistically significant difference. On the other hand, luliconazole 5% solution showed therapeutic effects on the 4- and 8-week infection models with 90% efficacy rates of 83% and 42%, respectively. Its efficacy rate in the 8-week infection model was significantly lower than that in the 4-week infection model. The 99% efficacy rates of luliconazole 5% solution in the 4- and 8-week infection models were both 0%. In a phase III study, efinaconazole 10% solution was reported to have a 28.8% complete cure rate in a subpopulation analysis of Japanese patients with moderate DLSO (infected area: 20–50%) [22]. In a postmarketing clinical study of Japanese patients, the complete cure rate of efinaconazole 10% solution was 25.0% for patients with including severe DLSO (infected area > 50%) [23]. Also, there are reports that efinaconazole 10% solution exerted clinical efficacy against refractory onychomycosis [24, 25]. Alternatively, luliconazole 5% solution was reported to have a 14.9% complete cure rate in a phase III study of Japanese patients with moderate DLSO (infected area: 20%–50%) [26]. However, there are no clinical studies on the use of luliconazole 5% solution in patients with more severe onychomycosis. Considering the above reports, efinaconazole 10% solution’s high efficacy in clinical studies reflects its efficacy even in more refractory 8-week model in this study.

The drug’s therapeutic efficacy is determined by its permeability and antifungal activity in the nails [16, 27]. In this study, luliconazole showed equal or higher nail concentrations than efinaconazole, presumably because of its higher keratin affinity. Still, both drugs were detected in the nails at significantly higher concentrations than the MECs in a keratin-added medium. Conversely, efinaconazole 10% solution was highly effective in reducing the nail fungal burden, whereas luliconazole 5% solution showed no 99% efficacy rate. Therefore, the effectiveness of efinaconazole was thought to be due to their different characteristics because efinaconazole has a lower keratin affinity and higher human nail permeability than luliconazole [16, 27]. In addition, our recent study showed that there was a drug concentration gradient from the superficial to the internal part of the treated nails, and in the deeper nail layer, the antifungal activity and the amount of permeated efinaconazole were higher than those of permeated luliconazole [27]. Considering these, it was believed that efinaconazole 10% solution was more effective than luliconazole 5% solution in reducing the nail fungal burden and its therapeutic efficacy was less influenced by the duration of infection, which is unlikely with that of luliconazole 5% solution, presumably because efinaconazole reached the nail’s deep layer and killed the infecting fungi.

In conclusion, an tinea unguium model close to a clinical infection condition was newly developed with fungal burden increased by extending the infection duration. Considering the increased infection intensity and intractableness to drugs and the relevance of clinical studies, the new tinea unguium model would predict the drug’s efficacy against moderate to severe onychomycosis, leading to the development of new topical anti-onychomycosis drugs.
